# Talent identification in soccer: The influence of technical, physical and maturity-related characteristics on a national selection process

**DOI:** 10.1371/journal.pone.0298359

**Published:** 2024-03-13

**Authors:** Michael King, Matthew Brown, John Cox, Ross McLellan, Christopher Towlson, Steve Barrett

**Affiliations:** 1 The University of the West of Scotland, Glasgow, United Kingdom; 2 Playermaker, London, United Kingdom; 3 University of Hull, Hull, United Kingdom; Instituto Politécnico de Santarém: Instituto Politecnico de Santarem, PORTUGAL

## Abstract

The present study examines the influence of technical, physical, and relative age characteristics on players selection success within the Scottish Performance School trials. Ninety adolescent players (81 males, 9 females; mean ± standard deviation: age = 11.3 ± 0.4 years, height = 149.6 ± 6.9 cm, mass 38.1 ± 4.7 kg) performed a battery of physical fitness (20m Sprint, CMJ, 5-0-5 agility test), anthropometric, and 8 small-sided games (SSG; 9v9) as part of a talent identification (TID) programme. Players technical (ball touches, time on the ball, high-speed releases) and locomotor activities (high-speed running distance, sprint distance, accelerations, and decelerations) were monitored using foot-mounted inertial measurements units during SSG’s. The data was analysed using independent sample T-tests. Mann-Whitney U analyses were conducted to examine the differences between groups whose data was determined as being (non)parametric, with Cohen effect sizes applied. Successful players performed significantly better during physical tests (Effect size ± confidence limits: Left 5-0-5 = -0.89±0.13, Right 5-0-5 = -0.51±0.11), had significantly higher locomotor activities during SSG (high-intensity distance = 0.4±26.6, horizontal accelerations = 0.59±1.19) and significantly higher technical outputs during SSG (touches = 0.71±6.1, releases = 0.49±2.5, high-speed releases = 0.59±2.7, time on the ball = 0.52±3.4) compared to unsuccessful players. Successful players had significantly higher locomotor activities and technical outputs during SSG than their unsuccessful counterparts. Monitoring technical and locomotor activities during SSG may compliment or replace physical testing batteries for assessing TID processes in soccer.

## Introduction

Soccer organisations utilise multi-faceted talent identification (TID) methods (physical, technical, psychological) to select the best players to enter their player development programmes [1]. Multiple key stakeholders (e.g., coaches, scouts, sport scientists) within organisations (e.g. professional football clubs or national governing bodies) utilise different methods of TID, such as subjective (e.g., perceived technical ability via scouting [2]) and objective measures (e.g., physical screening data [2]) of player performance, to identify male and female players who possess desirable characteristics to succeed within development programmes [1]. A consistent theme for identifying talented soccer players, is to perform a battery of tests which incorporate anthropometric, physical fitness and technical skill measures [1, 2]. The most common protocols observed in soccer include a physical testing battery (incorporating sprints, jumps, agility and anthropometric measures), with either full-sized or small sided games [3]. Given that adolescent soccer players are often undergoing a period of accelerated growth (typically referred to as peak height velocity [PHV] between ages 11 to 16 [4, 5]) TID assessments have been recommended to consider this as an influencing factor [6, 7].

Unlike professional clubs competing with one another for the best talent, where selection has shown maturity timing bias (i.e. early, on-time or late) [7–9], limited data has been presented on the maturity status of national level players, where the talent pool is limited by player eligibility. Given the presence of maturity selection bias within academy soccer [10, 11], it is important that the influence of growth and maturation are considered by practitioners when attempting to identify talented adolescent soccer players [12–14]. Chronologically age matched groups of academy soccer players, where children are grouped depending on their date of birth, often display large variations in anthropometric and physical fitness characteristics due to the non-linear relationship between physical development and age [15, 16]. Despite the fact that technical and psychological characteristics of players are considered key attributes for talent selectors [1], these maturity-related differences can lead to sub-conscious maturation selection bias [11], resulting in over-selection of early maturing players who possess temporary enhancements in maturity-related anthropometric (e.g., taller) and physical fitness characteristics (e.g., superior sprinting capacity) [17]. Soccer governing bodies already have a limited numbers of players to select from and a chronological age selection bias could result in a loss of talent where potentially less mature players are deselected based on their inferior physical attributes [7].

There are a number of different methods cited in the literature regarding how best to ensure ecological validity when practitioners, coaches, and scouts assess physical and technical capacity in adolescent footballers [18]. One way to improve the ecological validity of an assessment is to conduct it in a setting that is as similar as possible to the real-world setting in which the players perform. For example, if you are interested in assessing the technical skill of adolescent soccer players, you could conduct the assessment during a soccer practice or match rather than in a laboratory setting [19].

Therefore, the aim of the current study was to explore the technical, physical and locomotor traits of successful players during the national trial. The study hypothesis was that adolescent soccer players who successfully pass a trial process would exhibit superior physiological and technical characteristics compared to those who are unsuccessful [1, 2]. The findings of this study could allow for TID processes to become complimented and backed up by objective data in comparison to the current coach led, subjective, decision-making processes, within both closed (testing) and open (SSG) environments.

## Methods

### Participants

Ninety (81 male and 9 female), under-12 academy football players (mean ± standard deviation: age = 11.3 ± 0.4 years, height = 149.6 ± 6.9 cm, mass 38.1 ± 4.7 kg) from Scottish Football Association (S.F.A) registered clubs, were included in this study. The researchers had no control over the recruitment of any of the participants in the present study, as all players had been invited to the national trial by coaches and scouts who watch academy games on a regular basis. Following examination of generic performance-related characteristics in adolescent soccer, it has been argued that males and females should train and play together up until approximately 12 years of age in an attempt to maximise talent pools and increase skill development [20]. All players were free from injury and illness prior to the national trial and permission was granted, during registration, from parents/guardians for their data to be collected and analysed as part of the national trial process via written informed consent (ethical no. 16783). Data collection was part of the S.F.A regular monitoring procedures and conformed to the Declaration of Helsinki [21].

Talented soccer players as identified by the Scottish Football Association (S.F.A.) are selected and invited to attend a specific performance school within their geographic region which will facilitate additional systematic training (alongside training with their parent club) starting as young as 11 years old. Permitting young players, the opportunity to accumulate additional training hours is one of the fundamental concepts of the national performance soccer school programme [17]. To be successfully offered a place in the S.F.A. performance school programme, players are selected via a national trial. Following the national trial, players are either offered a place within the national performance soccer school programme (i.e., “successful”) or not (i.e., “unsuccessful”). Within this trial, players perform a battery of physical tests, coach-led technical practices, and small-sided games (SSG).

### Experimental design

The present study adopted a cross-sectional study design. The players’ selection status was decided upon by coaches’ and scouts’ subjective opinion where they were “successful” or “unsuccessful” in being offered a place in the performance school programme, following the national trial. No references or prior instruction was provided coaches and scouts in order to maintain the previous selection process and avoid bias towards the purpose of the present study.

### Inertial measurement units

Player technical and locomotor activities (total distance covered, high-speed running distance[>4m/s] [22], sprint distance [>5.5m/s], accelerations and decelerations[±2m/s/s] [23]) and technical actions (ball touches, time on the ball, high-speed releases [>15m/s] [24]) were quantified using commercially available foot-mounted, inertial measurement units (IMU`s) (PlayerMaker^™^, Tel Aviv, Israel). Each IMU incorporated two components from the MPU-9150 multi-chip motion tracking module (InvenSense, California, USA), being a 16 g triaxial accelerometer and a 2000°•sec^-1^ triaxial gyroscope. Housed in manufacturer-supplied tightly fitting silicone straps, each player was equipped with two IMUs (one for each foot), which were located at the lateral malleoli over the player’s boots. To diminish issues related to inter-unit reliability, players used the same IMUs throughout the data collection period [25]. The IMUs showed good intra-unit reliability (Proportion of agreement (PA) = 95.9% - 96.9%, Coefficient of Variance (CV) = 1.4% - 2.9%) and validity (PA = 95.1% - 100.0%) compared to retrospective video analysis [26]. All devices were activated via a Bluetooth connection to an iPad (Apple Inc, California) prior to each training session. Data were uploaded to the manufacturer’s cloud-based software (v.3.22.0.02) post-session by the national teams’ practitioners. The start and end of each trial was identified and tagged prior to data being exported from the manufacturer’s cloud-based software into Microsoft Excel 2020.

### Trial session content

**Fig 1:**
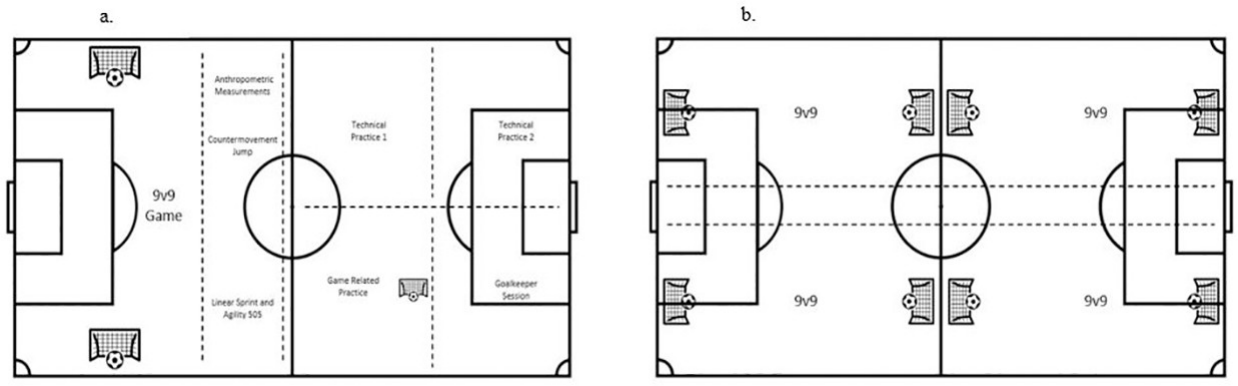
a and b represent the national trial morning session plan (a) and the national trial afternoon session plan (b).

Trial session activities were split into two sessions (morning and afternoon). In the morning, 8 stations were completed (Fig 1a). Trialists were split into 8 groups, where each group firstly completed a FIFA 11+ warmup and then commenced the carousel of 8 training stations, starting at a different station, and moving to each subsequent station in the same order. Five football specific stations (1. Two technical passing drills, 2. Technical finishing drill, 3. Team passing drill, 4. Two 9 vs 9 games with one goalkeeper per team) and 4 physical testing battery stations (1. 20-metre linear sprints; ICC = 0.81, 2. 5-0-5 Agility test; ICC = 0.99, 3. Countermovement jump; ICC = 0.88, 4. Anthropometric measurements; stature ICC = 0.89, seated stature ICC = 0.96 mass = 0.97) were completed by players. Players spent 10 minutes at each station in their groups and were permitted two minutes of rest in between each station. The morning session concluded once each group had taken part in each of the 8 stations once. Groups moved in a clockwise direction round the stations.

Following 60 min of passive recovery, the afternoon session comprised of the same 8 teams as the morning session, with teams playing in a 9v9 small-sided games tournament (Fig 1b). The 9v9 games were played on a pitch that was 52.5m long and 34m wide and lasted for 10 minutes per game. The games were officiated by qualified officials from the Scottish Football Association. There were no conditions placed on match-play and officials were asked to govern according to the general laws of the game. During match-play coaches were asked not to coach or verbally encourage players to avoid coaches potentially influencing the actions of players.

### Anthropometric measures

Using the International Society for the Advancement of Kinanthropometry (ISAK) guidelines [27], the stretch stature, sitting stature, and body-mass were measured using a portable stadiometer and scales, respectively (SECA, Hamburg Germany). All anthropometric data was collected by practitioners that were familiar with collecting this kind of data in a similar environment. Firstly, participants were asked to remove their shoes and stand on a calibrated floor scale to determine their body-mass (kg). Secondly, participants were asked to stand up straight, with their shoulders back, facing forward on a portable stadiometer to determine stature (cm). Lastly, a purpose-built box (33cm high) was placed on the base of the portable stadiometer, and participants were asked to sit on the box with their back resting flat against the back of the stadiometer, sit up straight, with their shoulders back–where sitting stature was measured. The estimation of biological maturity status was established using the Fransen method [28] by entering the players’ date of birth, stretch stature, sitting stature, body-mass and the test date into a purpose-built Microsoft Excel spreadsheet [12], which enters the data into a multiple regression equation and provides an estimation of the proximity to PHV expressed in years from PHV, with a score of “0” meaning an individual is at PHV. The equation for calculating maturity ratio using the Fransen method is as follows:

$$\begin{array}{l} =6.99+\left(0.116.CA\right)+\left(0.00145.CA2\right)+(0.00452\times Body\;Mass)\\ -(0.0000341\times Body\;Mass\;2\;16)-(0.152\times Stature)+(0.000933\times Stature\;2)\\ -(0.00000166\times Stature\;3\;17)+(0.0322\times Leg\;Length)\\ -(0.000269\times Leg\;Length\;2\;18)–(0.000761\times [Stature\times CA]) \end{array}$$

### Physical fitness measures

Linear maximum speed was estimated using electronic timing gates (Brower Timing Systems, Utah, USA) during 20-meter straight-line sprints. Out of three attempts for each test, the best counted. Three attempts were provided to allow players to familiarise themselves with the test and increase the likelihood of them performing to their maximum capability. Change of direction ability was assessed using the agility-505 test in line with Chaalali et al. (2016) [29]. Players were instructed to sprint 15m to a marked point then turn and sprint back to a point marked at 10m. Electronic timing gates (Brower Timing Systems, Utah, USA) were used during this assessment. Time to complete the turn was collected (2 x 5m, turning off both the left and right foot were taken, and an average time was used in final analysis). Each participant also performed three squat jumps [30] using an electronic jump mat (Just Jump, Probotics, Alabama, USA) to measure jump height.

## Statistical analysis

The physical fitness testing battery, technical data, and locomotor activities from SSG’s were presented as mean ± standard deviation (SD). Analyses were conducted to establish significant differences in physical fitness, technical measures, locomotor activities and maturity status between successful (selected) and unsuccessful (deselected) groups of players. Prior to analysis, data was checked for normal distribution using Shapiro-Wilk tests. Independent samples’ t-Test was used to determine differences between groups, and Non-parametric tests were applied where data did not met the assumption of normal distribution (Mann-Whitney U analyses). Statistical significance was set at p < 0.05. Effect sizes (ES) were calculated to determine the magnitude of the difference between groups, and were interpreted using Hopkin’s scale, where ≤ 0.2, 0.21–0.6, 0.61–1.20, 1.21–2.00 and 2.01–4.0 were interpreted as follows; trivial, small, moderate, large and very large differences, respectively [31].

## Results

### Anthropometric, age and maturity-related data

No significant differences in chronological age (Successful = 11.20±0.43 yrs., Unsuccessful = 11.3±0.44 yrs., p *=* 0.41), height (Successful = 149.90±7.10cm, Unsuccessful = 149.30±6.60cm, p = 0.72), weight (Successful = 39.00±5.20kg, Unsuccessful = 37.10±4.10, p = 0.32kg), and maturity offset (Successful = -2.02±0.51, Unsuccessful = -2.12 ± 0.51, p *=* 0.41) were observed between the Successful and Unsuccessful group.

### Testing battery

Successful (e.g., selected) players produced higher scores in the counter-movement jump (Mean ± SD: Successful = 40.0±5.84 cm, Unsuccessful = 37.7±3.98 cm, *p* = 0.186, ES = 0.18; *trivial*), and significantly quicker times within the 5-0-5 Agility test on the left (Successful = 2.47±0.11s, Unsuccessful = 2.57±0.13s, *p* = <0.001, ES = -0.89±0.49; *moderate*), and right sides (Successful = 2.49±0.11s, Unsuccessful = 2.54±0.10 s, *p* = 0.033, ES = -0.51±0.46; *small*) compared to unsuccessful players. Successful players also had quicker 20m sprint time (Successful = 3.59±0.16s, Unsuccessful = 3.65±0.13s, *p* = 0.127, ES = -0.21; *small*) compared to those who were unsuccessful (Table 1).

**Table 1:** Differences in test battery scores between successful and unsuccessful players, data distribution and normality, p-values and effect sizes with 95% CI.

Metric	Successful	Unsuccessful	Mean Difference	p-value between groups	Effect Size (95% CI)
	Mean ± SD (95% CI)	Mean ± SD (95% CI)			
Countermovement Jump (cm)	40.0 ± 5.84 (38.4–41.6)	37.7 ± 3.98 (36.3–39.2)	2.30	0.19 (Nonparametric)	0.18 (-0.72–4.49)
20m Sprint (secs)	3.59 ± 0.16 (3.54–3.63)	3.65 ± 0.13 (3.61–3.70)	0.06	0.13 (Nonparametric)	0.21 (-0.13–0.001)
Left 5-0-5 (secs)	2.47 ± 0.11 (2.44–2.50)	2.57 ± 0.13 (2.53–2.62)	0.10	<0.001***	-0.89 (-1.38 - -0.40)
Right 5-0-5 (secs)	2.49 ± 0.11 (2.46–2.52)	2.54 ± 0.10 (2.50–2.58)	0.05	0.033*	-0.51 (-0.97 - -0.04)

* Denotes p < 0.05,

*** Denotes, p < 0.001.

### Locomotor activities

**Fig 2:**
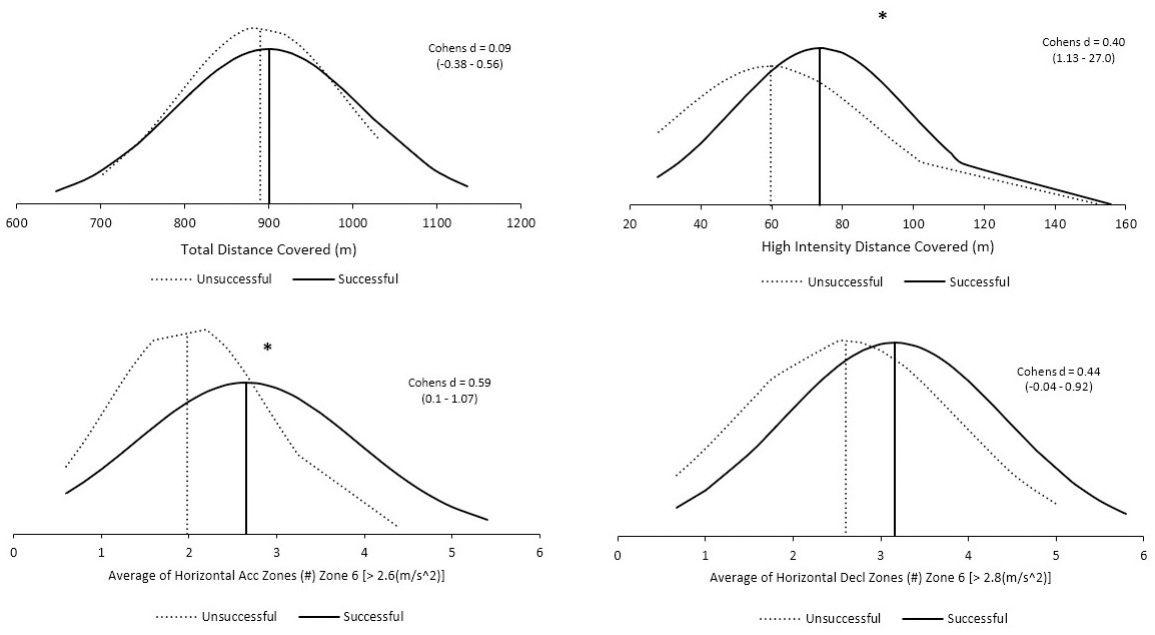
Comparison of unsuccessful vs. successful physical metrics during 9v9 match play. * Denotes p < 0.05.

Successful players produced greater physical outputs, on average, (Fig 2) with regards to Total Distance Covered (Successful = 900±113.6, Unsuccessful = 890.4±99.6m; p = 0.715; ES = 0.09; *trivial*), and number of Decelerations (Successful = 3.2 ± 1.3, Unsuccessful = 2.6±1.3; p = 0.07; ES = 0.44; *small*), compared to unsuccessful players. Successful players displayed significantly greater high-speed running distance covered (Successful = 73.7±24.8m, Unsuccessful = 59.6±27.8m; p = 0.004; ES = 0.40; *small*) and number of accelerations performed (Successful = 2.7±1.3m, Unsuccessful = 2.0±0.9m; p = 0.026; ES = 0.31; *small*) compared to unsuccessful players.

### Technical actions

**Fig 3:**
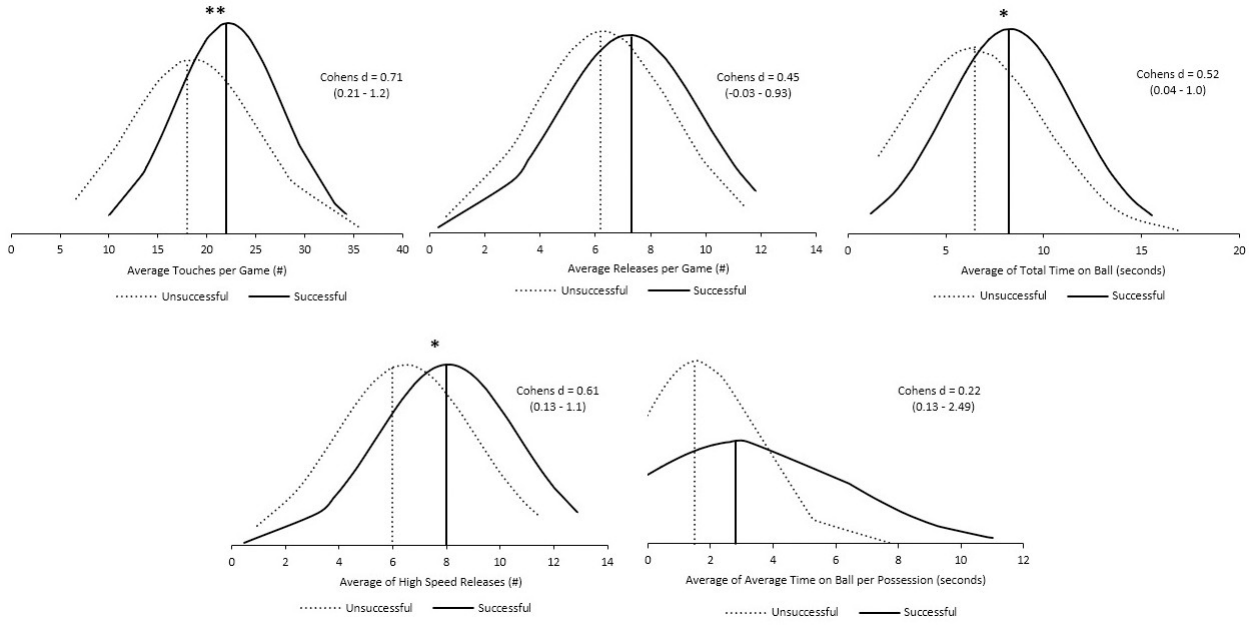
Comparison of unsuccessful vs. successful technical metrics during 9v9 match play. * Denotes p < 0.05, ** Denotes p < 0.01.

Successful players technical metrics were higher than the unsuccessful players, with significantly greater number of touches (Successful = 22±5, Unsuccessful = 18±7; p = 0.004; ES = 0.71; *moderate*), time on the ball (Successful = 8.2±3.1s, Unsuccessful = 6.5±3.6s; p = 0.033; ES = 0.52; *small*), and high-speed releases (Successful = 8±3, Unsuccessful = 6±3; p = 0.013; ES = 0.61; *moderate*) performed (Fig 3).

## Discussion

The aim of the present study was to explore the influence of technical, physical, and maturity-related characteristics on players selection success within the Scottish Performance School trials, as part of their TID process. The main findings of this study were two-fold: 1) Both open and closed environment TID processes showed the same outcomes for successful and unsuccessful players. That is, successful players displayed better physical and technical outputs compared to unsuccessful players. 2) No bias was evident between successful and unsuccessful players, during TID processes, with regards to anthropometric characteristics or estimation of peak height velocity. The present study is novel as technical skill and physical prowess have been quantified using match-play. Whereas, in existing research these attributes are often examined using closed tasks, for example a physical testing battery [14] or technical tests such as the Loughborough passing test [32]. Previously, quantifying technical competency has not been commonplace. Any existing research that has attempted to assess technical competency has relied on extensive video analysis procedures that are time consuming and require specialist support (e.g., performance analyst) [22, 33]. The current approach allows for coaches and scouts to identify talent using objective data in an environment that is more soccer specific, as opposed to isolating physical and technical performance using a closed testing battery.

TID processes often incorporate screening of players, focused on their ability to complete tests assessing their physical and technical capabilities [34, 35]. Within the testing battery performed, players who were “successful” at the national trial recorded better times for the 20m sprint test, 5-0-5 agility test and had a higher CMJ output. However, the use of closed tests have lacked the ecological validity of assessing players within their specific sport [14]. For example, Towlson and colleagues [35], have utilised various SSG’s to assess players technical, physical and subjective soccer abilities. Understanding how players perform in both open and closed environments can provide insights into a player’s current ability and also highlight potential development areas [14]. However, until recently, gaining ‘objective’ data from these open environments required manual coding of video using human resource time [22]. With the foot-mounted IMU’s utilised within the current study, objective data was automated about players performance within both the open and closed environments, reducing any potential human error as observed in previous video analysis literature [22]. Previous literature that has attempted to differentiate between elite and sub-elite adolescent soccer players has highlighted that there were clear advantages in using ecologically valid measurements to determine game specific skills, as opposed to closed skill tasks [36].

“Successful” players also produced greater in-game physical outputs with significantly greater high-speed running distance and number of horizontal accelerations performed during the 9v9 match format (games played during afternoon session). Generally, the “successful” group demonstrated the best physical performance in both the testing battery and in-game. This raises the question whether it is necessary to administer both. It may be more efficient and save time for future trials if any physical assessments were made using in-game data and the physical testing battery was removed from the trial day format completely. However, individual player-level analysis will need to be performed to understand if there were players who did not fit this trend. For example, players who may have performed well in both open (game play) and closed (test battery) physical tests but were ultimately “unsuccessful” in the trial. Likewise, are there players who performed well in one of the physical tests but not the other and what was their overall trial outcome? Uncertainty surrounding player outcomes following trials further bolsters support for a multidimensional approach to TID using both subjective and objective assessment [37].

Timing of the adolescent growth spurt has been shown to bias TID outcomes in previous soccer research [10, 11, 35]. Therefore, it is important to consider both growth and maturation within the development of TID and long-term soccer development programmes [4, 12, 14]. For example, in bio-banded small-sided games, Towlson and colleagues [11] looked at the passing network trends within youth players, showing that players at a higher maturity status, tended to have the ball passed to them more often. This may then have implications for coaches and scouts to have more opportunities to see these players during small-sided games, which could bias opinions [11]. While the current study did show no differences between groups for anthropometric, relative age effect or maturity status these findings should be interpreted with caution due to the methods used to quantify maturity status. Despite evidence to suggest that older, biologically more mature players are overrepresented in adolescent soccer programmes, the present study found that there were no significant between group differences in age, maturity offset values and anthropometric measures. With the coaches ‘blind’ to the objective data from the testing battery and SSG’s, coaches seemed unbiased by anthropometric characteristics and players maturity status, and instead focused on the technical and physical qualities of the players at the trial. Typically, during TID process in adolescent soccer there is a selection bias in favour of players with advanced maturity status [11], however, that does not seem to be the case in the present study. Coaches in the current set up are actively involved in trying to reduce maturity related selection biases (i.e., through coach education pathways) and the present study has provided evidence that this approach may be working. Unlike domestic TID research, the talent pool that these coaches can select from may be limited (e.g. players must be of Scottish ancestry), which further supports the importance of removing any possible maturity related selection bias. However, caution must be applied when making inferences surrounding maturation due to the amount of error in some of the methods used to estimate maturity status [38]. Further research is required to look at the entire selection process (as opposed to only the final national trial) across multiple age groups, to identify if this trend is consistent throughout the selection pathway. It is possible that existing pre-selection processes might have attenuated the effects within the sample between successful and unsuccessful players.

### Limitations

The present study was able to make some inferences based on maturity status. However, intimate physical examination [39] and/or x-ray evaluation of skeletal maturation are frequently the gold-standard techniques for determining maturation status [40]. Due to these procedures’ intrusive nature, adopting them in many situations is either inappropriate, unethical, or unfeasible. More pragmatic methods of skeletal age assessment such as ultrasound [41] were also deemed not cost effective. As a result, several methods for estimating maturity have been created. There appears to be consensus in the literature that the Khamis and Roche equation is the preferred method for estimating maturity status (by showing percentage of final estimated adult stature) [12], however, one issue that is apparent when attempting to use the equation in an applied setting is the need for measures of parental stature. It was not possible to obtain parental stature during the current project and therefore the Fransen et al. [28] prediction equation was chosen to calculate maturity offset because it is non-invasive, economical, and quick. As a result, field-based research like ours frequently use similar techniques [42–45]. While valid when applied closer to PHV, equation-based estimates of maturity offset have limitations in early- and late-maturing individuals, as well as when used prior to the age of 11 [12]. The most practical choice in youth soccer situations, however, is to use non-invasive estimations of somatic age by anthropometric measurement due to the limitations of conducting radiographic assessments of skeletal maturity.

Additionally, although the research team had no influence of the participants involved in the national trial it must be acknowledged that the inclusion of only nine female participants may have had an influence on results. It would be useful in future research to examine male and female players in separate settings.

Another potential limitation of the study was the lack of standardisation in the experience and qualifications that coaches and scouts who selected players had. Broadly speaking, head coaches were qualified to a minimum of UEFA A License, and scouts would have completed a minimum of the SFA Talent Identification course (Level 1). Additionally, coaches and scouts were using a 4-corner model of “technical/tactical”, “psychological”, “physical” and “social” as a bases to select players. However, an in-depth analysis of this model and its application on the day of the national trial was outside the scope of this study.

It may have been beneficial to examine other potential predictors of “success” on the day of the national trial. Additional physical measures would have been of interest such as ability to repeat high-intensity running (measured using a yo-yo test) or measures of flexibility (such as a straight leg raise). Additionally, future studies may wish to consider using video analysis to add further context to the technical characteristics that were collected at the national trial. Unfortunately, the inclusion of video analysis was outside the scope of this study due to time and financial constraints.

### Practical implications

The present study holds important applied implications for practitioners, coaches and scouts involved in TID processes. Firstly, it appears that stakeholders involved with the cohort can differentiate between different levels of physical and technical prowess during a national trial, irrespective of maturity status, without use of the data that was collected. It is possible that rigorous coach education that focuses on mitigating the profound impact that biological maturation can have on TID procedures has influenced decision making [46]. Implementation of such coach education should be considered in clubs and at national associations. Secondly, it is important to note that players who displayed better physical performance in the isolated test battery also showed greater physical characteristics during match-play. The design of future TID procedures should be reviewed to consider the need for isolated physical test batteries. If physical performance can be readily examined in a more realistic environment (match-play) then it would seem appropriate to do so. Additionally, research has shown that technical skills such as dribbling, passing, juggling, shooting can be more indicative of long-term success, in adolescent soccer players, than measures of physical capacity such as a 40m sprint, a test of agility and a countermovement jump assessment [47].

## Conclusion

In conclusion, the present study has shown that soccer players who were “successful” in being selected at a national trial possessed greater physical and technical attributes compared to “unsuccessful” players. Players were deemed “successful” or not without sight of the data collected in the present study, suggesting that coaches and scouts appear to capable of subjectively selecting players who perform best from a physical (both in an isolated test battery and in-game) and technical standpoint. Technical and physical differences appear to be independent of maturity status, however, caution should be applied during the interpretation of such findings due to the error associated with estimates of biological maturation.
